# Comparative transcriptomics revealed parallel evolution and innovation of photosymbiosis molecular mechanisms in a marine bivalve

**DOI:** 10.1098/rspb.2023.2408

**Published:** 2024-05-29

**Authors:** Ruiqi Li, Daniel Zarate, Viridiana Avila-Magaña, Jingchun Li

**Affiliations:** ^1^ Ecology and Evolutionary Biology, University of Colorado Boulder, Boulder, USA; ^2^ Museum of Natural History, University of Colorado Boulder, Boulder, USA

**Keywords:** photosymbiosis, molecular mechanisms, Fraginae, transcriptome, parallel evolution, mollusc

## Abstract

Photosymbioses between heterotrophic hosts and autotrophic symbionts are evolutionarily prevalent and ecologically significant. However, the molecular mechanisms behind such symbioses remain less elucidated, which hinders our understanding of their origin and adaptive evolution. This study compared gene expression patterns in a photosymbiotic bivalve (*Fragum sueziense*) and a closely related non-symbiotic species (*Trigoniocardia granifera*) under different light conditions to detect potential molecular pathways involved in mollusc photosymbiosis. We discovered that the presence of algal symbionts greatly impacted host gene expression in symbiont-containing tissues. We found that the host immune functions were suppressed under normal light compared with those in the dark. In addition, we found that cilia in the symbiont-containing tissues play important roles in symbiont regulation or photoreception. Interestingly, many potential photosymbiosis genes could not be annotated or do not exhibit orthologues in *T. granifera* transcriptomes, indicating unique molecular functions in photosymbiotic bivalves. Overall, we found both novel and known molecular mechanisms involved in animal-algal photosymbiosis within bivalves. Given that many of the molecular pathways are shared among distantly related host lineages, such as molluscs and cnidarians, it indicates that parallel and/or convergent evolution is instrumental in shaping host–symbiont interactions and responses in these organisms.

## 1. Introduction

Among the diverse symbiotic associations found in nature, photosymbiosis is both evolutionarily prevalent [1,2] and ecologically significant [3]. This association involves nutrient exchange between heterotrophic hosts and photosynthetic symbionts, where the symbionts provide photosynthates to the hosts and gain inorganic nutrients and shelter in return [2]. Photosymbioses have independently evolved in many host groups, including single-celled eukaryotes and animals like cnidarians and molluscs [4,5]. These associations play important roles in Earth’s primary production and biogeochemical cycling and are crucial in supporting the integrity of marine ecosystems [3,6]. Despite the ecological importance of photosymbioses, our knowledge of their diversity and molecular mechanisms remains rudimentary [7]. Given the prevalence of photosymbioses in drastically different eukaryotic lineages, it is important to study non-cnidarian hosts to gain comparative perspectives and to elucidate what evolutionary mechanisms drove the independent evolution of photosymbioses.

Two extant bivalve lineages have established obligate photosymbiosis with dinoflagellate algae from the family Symbiodiniaceae: the giant clams (Tridacninae) and the less-studied heart cockles (Fraginae) [1,4]. Like corals, photosymbiotic bivalves serve as keystone species in their ecosystems by providing structural complexity [8,9] and facilitating environmental carbon and nutrient cycling [10]. Several studies have started to explore molecular pathways underpinning bivalve photosymbiosis. Some genes involved in cnidarian photosymbiosis, such as carbonic anhydrase and vacuolar-type H^+^-ATPase (VHA), play similar roles in bivalves [11–13]. Given that bivalves harbour photosymbionts extracellularly, which is structurally and physiologically different from intracellular cnidarian photosymbiosis, it is intriguing that these distantly related host species adopt similar genetic mechanisms for photosymbiosis. It raises fundamental questions about the evolution of similar symbiotic relationships across different species. How do these diverse organisms, with their unique physiological setups, navigate the complexities of symbiotic interactions? Do bivalves and cnidarians generally use the same genes for symbiont–host interactions? Do bivalves evolve unique molecular adaptations for extracellular photosymbiosis? To answer these questions, a comprehensive assessment of bivalve photosymbiosis molecular pathways is needed.

The bivalve subfamily Fraginae provides an ideal system to investigate molecular mechanisms of photosymbiosis because it is composed of two sister clades, one exclusively photosymbiotic and one non-symbiotic [4]. This provides us with an opportunity to compare gene expression patterns of species with different lifestyles and discover potential molecular mechanisms in photosymbiosis. In this study, we used transcriptome profiles of a photosymbiotic species (*Fragum sueziense*) and a non-photosymbiotic species (*Trigoniocardia granifera*) to investigate molecular mechanisms in bivalve photosymbiosis.

**Figure 1.**
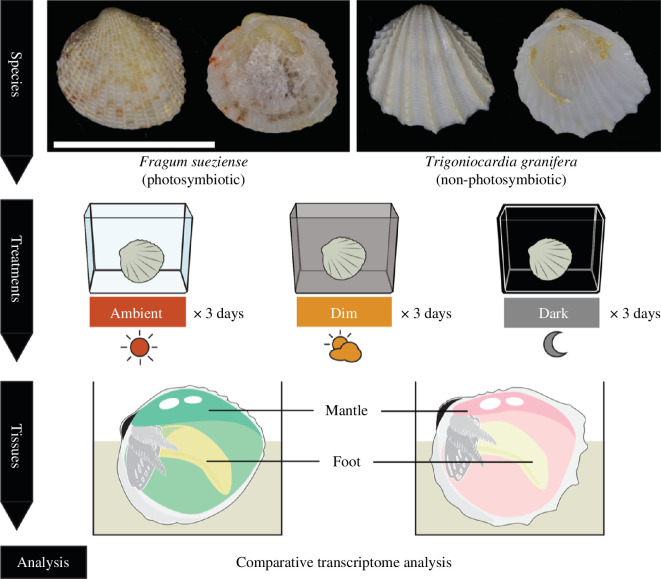
Experimental design. *Fragum sueziense* and *T. granifera* were randomly assigned into three groups evenly. These three groups were placed under different light intensities: one under an ambient light–dark cycle, one at a reduced light–dark cycle and the other under constant darkness. Specimens were treated for 3 days. RNA from the mantle (symbiont-hosting in *F. sueziense*) and foot tissues of all samples were extracted and then sequenced.

*Fragum sueziense* is a widely distributed species across the tropical Indo-Pacific Ocean. It hosts symbionts (Symbiodiniaceae: *Cladocopium*) extracellularly in a tubular network in the mantle and gill tissues, while the foot tissue is largely symbiont-free (figure 1). This allows us to explore gene expression profiles in symbiont-containing and symbiont-free tissues. As a contrast, we also investigated gene expression of a non-photosymbiotic Fraginae species *T. granifera* (figure 1), which is a heterotrophic filter feeder distributed in the tropical eastern Pacific.

We subjected both species to ambient, reduced and no light treatments (figure 1), assuming that photosymbiosis functions normally under ambient light and is slightly disrupted under reduced light and is shut down during prolonged darkness. Genes that are potentially involved in key photosymbiotoic interactions may exhibit the following expression patterns: (i) in *F. sueziense* mantle (symbiont-containing) tissues, the gene should be highly expressed in ambient light, downregulated in reduced light and minimally expressed in the dark, (ii) it should neither be highly expressed nor show an expression gradient in response to light in the *F. sueziense* foot (symbiont-free) and (iii) the gene should not be expressed in the non-symbiotic species *T. granifera*, or its expression level should not show a gradient with respect to light.

In addition to the top-down approach, we also paid special attention to several important metabolic pathways known to play crucial roles in stable photosymbiosis. These included carbon concentration mechanisms, carbohydrate metabolism [13], lipid metabolism and signalling [14,15], as well as nitrogen transportation [16]. We validated if genes involved in these processes were found in the bivalve transcriptomes and if they fit the expression criteria described earlier. Overall, this comparative transcriptomics approach allowed us to discover important and potentially novel molecular mechanisms in animal photosymbiosis.

## 2. Material and methods

### (a) Experimental design

*Fragum sueziense* was collected in Family Beach, Guam, and *T. granifera* was collected near Isla Venado, Panama. Thirty *F*. *sueziense* and 37 *T*. *granifera* were randomly assigned into three groups evenly and placed under different light intensities: (i) under an ambient light–dark cycle, which the animals were exposed to in the natural habitats, (ii) at reduced light during the day by blocking sunlight with black meshes and (iii) under constant darkness by blocking sunlight with multiple layers of black meshes (figure 1). Specimens were treated for 3 days and then processed. See detailed collecting information in the electronic supplementary material text and electronic supplementary material, table S1.

### (b) Transcriptome sequencing

Total RNA from the mantle and foot tissues of the two species were extracted. For *F. sueziense*, foot tissue from 4 to 5 individuals in the same treatment was grouped together for extraction due to their small sizes. RNAseq was conducted by Novogene (Novogene Corporation Inc., USA) following the manufacturer’s protocols (electronic supplementary material, text).

(c) Assembly, filtering and annotation

We first assembled one meta-transcriptome (including all treatments and tissue types) for each bivalve species (electronic supplementary material, text). Because the *F. sueziense* meta-transcriptome included reads from both the host and the symbiont, contigs were mapped to a custom *F. fragum* and *F. whitleyi* genome database and a custom Symbiodiniaceae database (electronic supplementary material, file S1), respectively. Contigs whose best hit mapped to the Symbiodiniaceae genome were removed. For both *F. sueziense* and *T. granifera* meta-transcriptomes, contigs that mapped to the Fraginae ribosomal RNA [4] were also removed. The meta-transcriptomes were annotated with eggnog mapper [17] with default settings.

### (d) Gene expression analyses

Transcript abundances were estimated using the alignment-free method kallisto v.0.46.2 [18]. Trimmed reads from individual samples were mapped to the *F. sueziense* or *T. granifera* meta-transcriptome, respectively. Transcript counts were normalized with the trimmed mean of M-values (TMM) method [19]. Principle component analyses (PCA) were conducted in R 4.2.2 to assess variations in overall gene expression among different species, tissues and treatments. Heat maps and dendrograms were generated to visualize the clusters of individual samples. For both bivalve species, we assessed differential gene expression (i) between mantle and foot tissues under ambient conditions and (ii) among mantle tissues across the three treatment conditions. Differentially expressed genes (DEGs) were identified with DESeq2 v.1.38.3 [20]. Any gene expression below 5 transcripts per million (TPM) across all samples was discarded. We used a *p*-value of 0.05 and a log fold change of 1 as cut-offs when identifying the DEGs.

### (e) Functional enrichment analysis

Two functional enrichment analyses were conducted for both tissue types in the two species. First, to assess functional differences among symbiont-hosting and symbiont-free tissues, as well as functional dissimilarities under different treatments, we conducted gene ontology (GO) enrichment with the package GO_MWU [21] using rank-based two-tailed Mann–Whitney *U*-tests. For both species, we identified enriched GO terms in mantle and foot tissues under ambient conditions, as well as enriched GO terms in mantle tissues from pairwise comparisons of the three treatments. Enriched GO terms were further analysed with a custom R script in three categories: biological process (BP), cellular component (CC) and molecular function (MF).

Furthermore, we used overrepresentation tests with the topGO R package v.2.52.0 to visualize representative functions in the mantle and foot in the two species. Enriched BP GO terms related to DEGs in ambient conditions for each tissue type were clustered with a threshold greater than 0.7 for similarity. The results were manually curated and visualized with tree maps using REVIGO [22].

### (f) Identification of photosymbiosis-related genes

We generated several lists of genes that are potentially involved in photosymbiosis based on their expression patterns. First, we used OrthoFinder v.2.27 [23] to identify orthogroups between *F*. *sueziense* and *T. granifera*, and retained genes that were successfully assigned to orthogroups. For *F. sueziense*, genes showing upregulation in the mantles under ambient conditions (normal photosymbiosis) compared with total dark (photosynthetic shut down) were retrieved from the DEG analyses (unfiltered list 1). Genes upregulated within mantle tissues (symbiont-bearing) compared with foot tissues (no symbionts) under ambient conditions were identified as a second gene set (unfiltered list 2). Subsequently, genes were excluded from unfiltered lists 1 and 2 if their orthologues exhibited similar expression patterns in the non-symbiotic species *T. granifera* because similar expression patterns in the non-symbiotic species indicated that these genes were more likely to be involved in light–dark regulation or mantle-/foot-specific functions, instead of photosymbiosis. After exclusion, the intersection of lists 1 and 2 was identified as list 3.

Moreover, *F. sueziense* genes that were not assigned to orthogroups in the first step could represent novel genes associated with photosymbiosis. Therefore, the same approaches that generated unfiltered lists 1 and 2 were performed on the unassigned genes in *F. sueziense*. The intersection of the two sets of genes was grouped into list 4. Boxplots showing expression levels of lists 3 and 4 genes were generated using the ggplot2 package in R.

Genes that showed a reverse expression gradient to light may also play important roles in photosymbiosis. However, it was difficult to tease them apart from genes involved in dark stress (e.g. starvation) responses in *F. sueziense*. We, therefore, focused on genes with enhanced expression under increased light levels in this study.

Finally, photosymbiosis-related genes from other photosymbiotic invertebrates (bivalves and cnidarians) were identified from literature (electronic supplementary material, file S2). These genes were retrieved from *F. sueziense* and *T. granifera* transcriptomes using BLAST [24] search with a custom script. Fold changes of mantle gene expression levels from paired comparisons of the three light treatments were visualized with the ggplot package in R.

## 3. Results

### (a) Meta-transcriptome assembly and annotation

Meta-transcriptomes quality for *F. sueziense* and *T. granifera* are shown in table 1 and electronic supplementary material, text, electronic supplementary material figure S1 and file S1. Among the assembled contigs, approximately 55.4% and 51.2% were successfully annotated for *F. sueziense* and *T. granifera* (electronic supplementary material, table S2).

**Table 1.** Statistics of the meta-transcriptome. The abyss-fac tools were used to assess the final transcriptomes.

	***F. sueziense***	***T. granifera***
number of contigs	75 300	1 19 250
shortest (bp)	255	255
longest (bp)	28 689	35 148
N50 (bp)	1212	969
L50	14 640	20 499
GC content	42.6%	47.9%
sum (MB)	64.09	87.53
complete BUSCO	96.8%	96.7%

### (b) Gene expression analyses

**Figure 2.**
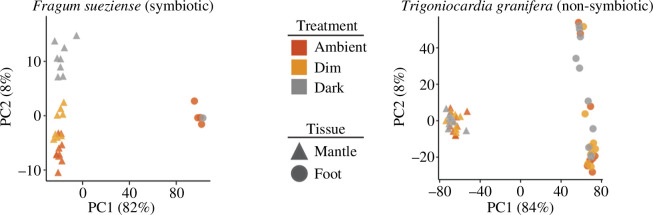
PCA plots visualizing overall gene expression variations of all samples. Both species exhibited clear separation between mantle and foot gene expression. *Fragum sueziense* mantle gene expression also clustered based on the three light treatments.

Overall gene expression-level variations from different species, treatments and tissue types are shown on the PCA plot (figure 2) and the heatmaps (electronic supplementary material, figure S2). PC1 explained 82% and 84% of the variance for the two species, respectively, corresponding to a clear mantle-foot separation. In *F. sueziense*, mantle transcriptome profiles separated based on the three light treatments along PC2, while this pattern was not found in the foot transcriptomes. Neither mantle nor foot clustered based on treatment groups in *T. granifera*.

**Figure 3.**
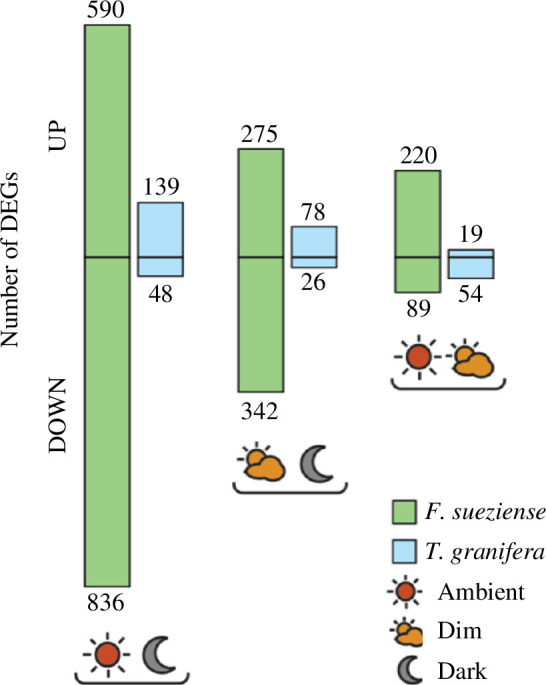
Bar plots reveal a higher number of DEGs among light treatments in *F. sueziense* compared with *T. granifera*.

We compared DEGs between the two tissue types under the ambient conditions for the two species, respectively. We also assessed DEGs across the three light conditions in the mantle tissues only (figure 3, electronic supplementary material, table S3). For both bivalve species, the largest number of DEGs (5722 for *F. sueziense* and 9760 for *T. granifera*) was found in the comparison between mantle and foot. In *F. sueziense*, the biggest difference in mantle gene expression comparisons was found between ambient and dark conditions (1426 DEGs). In *T. granifera,* however, considerably fewer genes (73–187) were found to be differentially expressed within mantles across the three light treatments.

### (c) Functional enrichment

**Figure 4.**
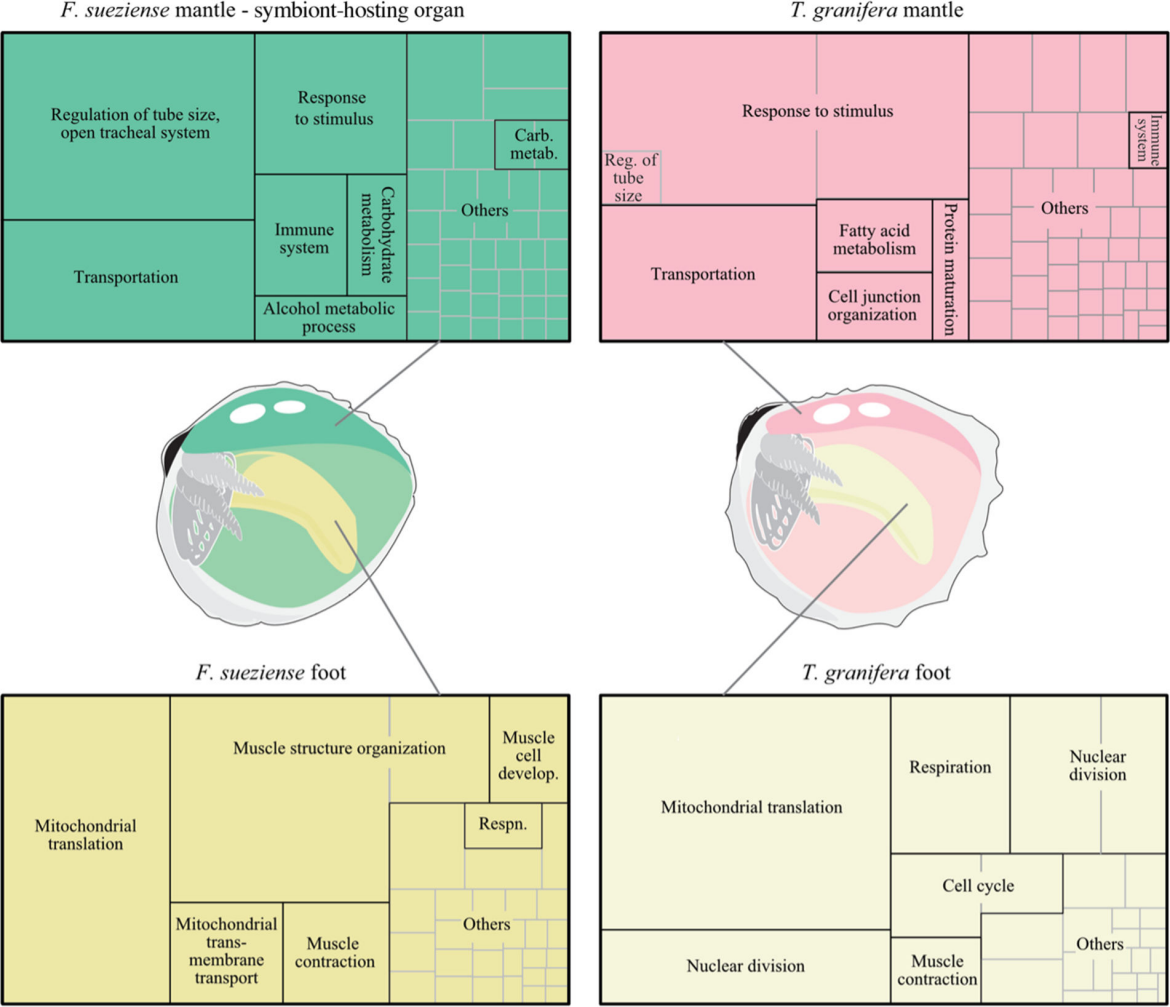
Enriched BP GO terms associated with upregulated genes in the mantles and foot of the two species. The tree maps provide valuable insights into non-redundant GO terms that are statistically enriched among the genes specifically upregulated in the mantle and foot of the two species, respectively. Each rectangle section corresponds to a non-redundant GO term, and the sizes of the rectangles are proportional to statistical significance measured by the log10-transformed adjusted *p*-values.

Functional enrichment analyses provided valuable insights into functional similarities of the same tissue types between symbiotic and non-symbiotic cockles. For both species, the same tissues exhibit some similar representative GO terms despite having distinct symbiotic statuses (figure 4 and electronic supplementary material, figure S3). The mantles displayed enrichment in functions such as maintaining an open tracheal system, facilitating transportation, participating in immune responses and being involved in various metabolic processes. In the foot, there was significant enrichment in functions associated with mitochondrial activities, respiratory processes and muscle development (figure 4).

**Figure 5.**
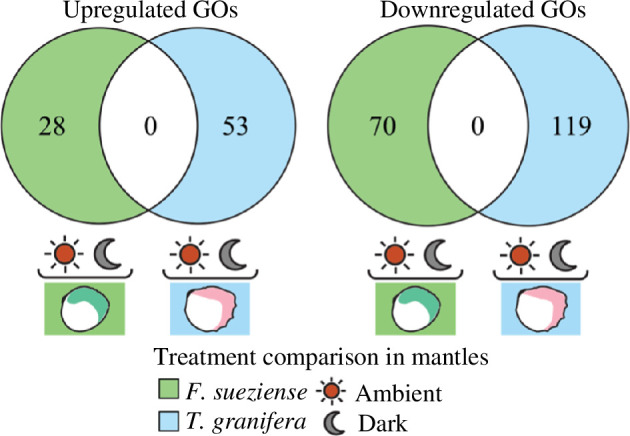
Venn diagrams illustrate that there were no overlapping GO terms between the mantles of the two species in the ambient-dark gene expression comparison.

Despite the tissue functional similarly, the two species exhibited distinct reactions to light. For *F. sueziense*, GO_MWU enrichment analysis revealed a significant enrichment of downregulated immune-related functions under ambient conditions when compared with dark conditions in the mantles. These functions encompass macroautophagy, the interleukin-1-mediated signalling pathway, the positive regulation of I-kappaB kinase/NF-kappaB signalling and so on. Moreover, several GO terms associated with transportation and metabolism, such as the regulation of monoatomic anion transport and the glutamine family amino acid metabolic process, were found to be enriched among the downregulated genes (table 2). It demonstrated a widespread upregulation of genes related to cilia and microtubules, including terms such as cilium, dynein complex and motile cilium (table 3). In the non-symbiotic cockle *T. granifera*, this comparison did not reveal any enrichment of these GO terms (figure 5).

**Table 2.** Selected downregulated GO enrichment results (*p* < 0.05) in the BP from GO_MWU in the mantles of *F. sueziense* in the ambient condition compared with in the darkness. In the non-symbiotic cockle *T. granifera*, the same comparison did not yield any enrichment of these terms.

	**t**erm	**n**ame
immune system	GO:0002253; GO:0002757; GO:0002764	activation of immune response
	GO:0002684	positive regulation of immune system process
	GO:0002699	positive regulation of immune effector process
	GO:0002702; GO:0002700	regulation of production of molecular mediator of immune response
	GO:0050778	positive regulation of immune response
	GO:0000423	mitophagy
	GO:0010508	positive regulation of autophagy
	GO:0016236	macroautophagy
	GO:0061912	selective autophagy
	GO:0070498	interleukin-1-mediated signalling pathway
	GO:0070555; GO:0071347	response to interleukin-1
	GO:0043123	positive regulation of I-kappaB kinase/NF-kappaB signalling
	GO:0002753	cytosolic pattern recognition receptor signalling pathway
transport	GO:0032892	positive regulation of organic acid transport
	GO:0044070	regulation of monoatomic anion transport
	GO:0051952	regulation of amine transport
metabolism	GO:0006103	2-oxoglutarate metabolic process
	GO:0006412; GO:0043043; GO:0006518; GO:004364	peptide metabolic process
	GO:0006520	amino acid metabolic process
	GO:0009064	glutamine family amino acid metabolic process
	GO:0009072	aromatic amino acid metabolic process
	GO:0009267; GO:0042594	response to starvation
	GO:0034224; GO:0120127	cellular response to zinc ion starvation

**Table 3.** Selected upregulated GO enrichment results (*p* < 0.05) in the BP from GO_MWU in the mantles of *F. sueziense* in the ambient condition compared with in the darkness. In the non-symbiotic cockle *T. granifera*, the same comparison did not yield any enrichment of these terms.

	term	name
cilia	GO:0001539; GO:0060285; GO:0030317; GO:0060294; GO:0097722	cilium-dependent cell motility
	GO:0003341	cilium movement
	GO:0042073	intraciliary transport
	GO:0030031; GO:0044782; GO:0060271; GO:0120031	cell projection assembly
	GO:0000226	microtubule cytoskeleton organization
	GO:0001578; GO:0035082	microtubule bundle formation
	GO:0007017	microtubule-based process
	GO:0007018	microtubule-based movement
	GO:0036158	outer dynein arm assembly
	GO:0070286	axonemal dynein complex assembly
	GO:0120036	plasma membrane-bounded cell projection organization
metabolism	GO:0033344	cholesterol efflux
	GO:0034381; GO:0097006	regulation of plasma lipoprotein particle levels
	GO:0034383	low-density lipoprotein particle clearance

### (d) Identification of photosymbiotic-related genes

**Figure 6.**
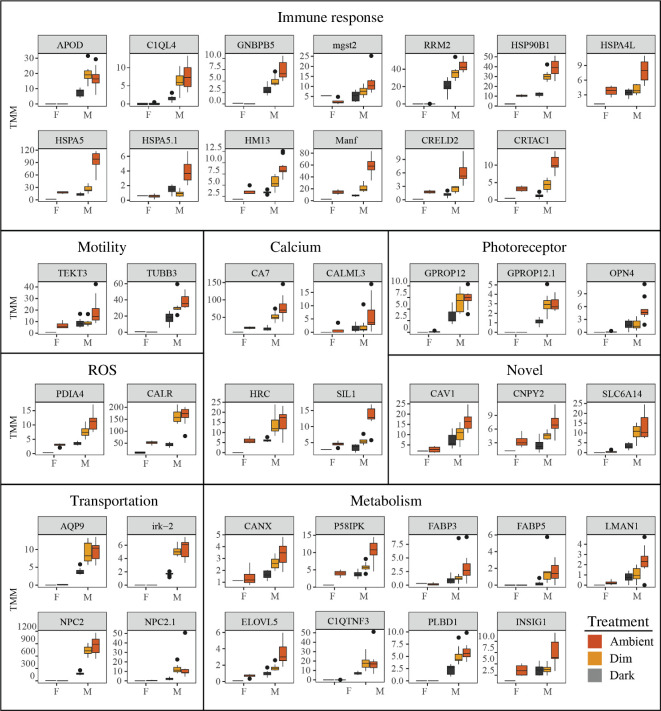
Box plots visualizing expression levels of selected genes from lists 3 and 4 in the mantle and foot of *F. sueziense*. Lists 3 and 4 refer to the intersection of upregulated mantle genes in the ambient-dark comparison and in the mantle-foot comparison (ambient) in *F. sueziense*. List 3 specifically excluded orthologous genes that exhibit upregulation in the same comparisons in *T. granifera*, while list 4 genes were not assigned to orthogroups. The selected genes, which were likely to play a role in photosymbiosis, were further classified based on their functions.

Regarding the photosymbiosis-related genes, there were a total of 140 genes categorized in list 3 (assigned to orthogroups) and 141 genes in list 4 (not assigned to orthogroups). Interestingly, genes lacking orthologues in their non-symbiotic relatives exhibited a lower annotation rate: 52.9% of genes from list 3 were annotated compared with 37.6% in list 4 (electronic supplementary material, table S2). The annotation of these genes revealed their association with crucial molecular photosymbiosis-related processes such as the immune response, metabolism, photoreception and reactive oxygen species (ROS) reactions (figure 6, electronic supplementary material, figures S4 and S5). Some immune-related enzymes (HSPA5, etc.) had clear expression gradients from dark to ambient light in the mantle tissues (hosting symbionts) but had zero or low expression levels in the foot tissues (no symbionts). Similar patterns were also found in other genes, such as carbonic anhydrases related to carbon-concentrating mechanisms (CCMs) [25], as well as Opsin 4 (OPN4) involved in light sensation (figure 6).

A total of 47 genes related to photosymbiotic mechanisms in other host organisms (giant clams, anemones and corals) were targeted (electronic supplementary material, file S2). The majority of them (39 and 43, respectively) were successfully matched with at least one orthologue. Among these genes, different expression trends across the three light conditions were observed (electronic supplementary material, figure S6, table S4 and file S2). Six isoforms from four genes showed expression patterns that fit our photosymbiosis gene criteria and were independently identified in lists 3 and 4. Those genes were related to cholesterol transportation, carbon concentration mechanism and symbiont recognition.

Interestingly for *F. sueziense*, we were either unable to retrieve any orthologue or found no trends in the retrieved orthologues of the light-enhanced calcification genes from giant clams [13], despite giant clams being phylogenetically closely related to Fraginae. Furthermore, 15 genes related to host-dependent nitrogen recycling and symbiont control in *Exaiptasia* [16] were targeted in *F. sueziense*. Among them, four genes were not retrieved from the BLAST search, five genes had no expression trends and three showed downregulation in ambient light conditions. Finally, genes related to symbiont phagocytosis in corals, such as LAMP1 [7], were not found in *F. sueziense* or *T. granifera*.

## 4. Discussion

### (a) Transcriptomic profile of *F. sueziense* mantle is highly influenced by the existence of symbionts

The bivalve mantle, a complex structure comprising multiple tissue types located beneath the shell [26] and often exposed to the environment, has evolved various adaptations to the organism’s lifestyle [27]. For example, in oysters and mussels, mantle mucosal secretion plays an important role in host defence [28]. The mantle can recruit haemocytes during infection, secrete bioactive molecules and show the melanization response [28,29]. In photosymbiotic giant clams (Tridacninae), iridocytes present in the mantles can manipulate light, promoting scattering of photosynthetically productive radiation (PAR) and reflecting non-productive wavelengths, therefore, safeguarding both hosts and symbionts from photodamage [30]. These demonstrate that the bivalve mantle is a crucial organ for interacting with environmental and endosymbiotic microorganisms.

The mantle tissues of *F. sueziense* and *T. granifera* exhibit enrichment of GO terms (compared with the foot) related to transmembrane transporters, immune functions, tube system development, sensory organ development and lipid metabolism (figure 4). This similarity is not surprising, as both species dwell in tropical shallow waters and share a semi-epifaunal filter-feeding lifestyle. These shared biological functions provide insights on what pre-existing pathways in the Fraginae common ancestor might be co-opted for establishing a photosymbiotic lifestyle. In the symbiotic *F. sueziense*, a greater number of enriched metabolic processes have been identified, particularly those associated with glycerolipids, cholesterol and glycoproteins (electronic supplementary material, table S5). These metabolic processes may already exist in non-symbiotic ancestors and have been co-opted in facilitating nutrient exchange in photosymbiotic species. Moreover, some enriched GO terms in *F. sueziense* might be associated with symbiont regulation, such as the regulation of the ERK1 and ERK2 cascade, which is involved in cell proliferation and apoptosis [31].

Furthermore, results from the light–dark experiment show that the existence of symbionts has a profound effect on the overall host mantle gene expression. In the non-symbiotic species *T. granifera*, mantle gene expression does not differ significantly under the three light conditions, while *F. sueziense* exhibits a much more pronounced response to dim and dark conditions (figure 3). For example, the GO term ‘response to starvation’ was enriched in the symbiotic cockles under darkness. In contrast, there were no indications of starvation in the non-symbiotic cockles when subjected to darkness (table 2). This suggests that photosynthetic energy plays a crucial role in the metabolism of Fraginae hosts, which is consistent with previous isotopic studies demonstrating that a portion of the organic carbon in Fraginae cockles is derived directly from the symbionts [32].

The impacts of symbionts on host gene expression have been documented in various photosymbiotic systems. For example, significant differences in host gene expression profiles between the symbiotic and aposymbiotic states were detected in a sea anemone (*Anthopleura elegantissima*). One of the key differential gene expressions is related to cell cycle progression and apoptosis [31]. In another photosymbiotic anemone (*Exaiptasia*), the host also exhibits modified gene expressions associated with growth, proliferation and nutrient transportation when in the symbiotic state [33,34]. Based on our findings, it can be inferred that while mantles and foot in the Fraginae cockles continue to perform their typical functions seen in other bivalves, the mantles of symbiotic cockles exhibit additional gene expression changes to accommodate and support the symbionts.

### (b) Photosymbiosis molecular mechanisms in Fraginae are shared with other host taxa

Some molecular mechanisms underlying Fraginae photosymbiosis are akin to those found in other photosymbiotic organisms [7]. For instance, we revealed suppression of immune-related genes in Fraginae symbiont-bearing tissues (table 2), aligning with findings in corals and sea anemones [35]. Some photosymbiosis genes revealed by this study have also been reported in other systems, encompassing recognition and regulation of symbionts, and nutrient exchange between hosts and symbionts.

For example, the cholesterol-transporter Niemann-Pick Type C2 (NPC2) was observed to be upregulated in *F. sueziense* mantles under ambient light (figure 6); and this pattern was not observed in the foot or in *T. granifera*. In mammals, NPC2 is responsible for exporting sterols from the lumen of late endosomes and lysosomes. In the Cnidaria-dinoflagellate symbiosis, NPC2 is among the highly upregulated genes in symbiotic *Exaiptasia pallida* and *Anemonia viridis* [36]. A specific homologue NPC2-d is closely associated with symbiosome membranes and facilitates the transfer of sterols from the symbiont to the host [14,37]. Based on the observed expression profiles, it is likely that NPC2 plays a similar role in sterol trafficking in the Fraginae cockles.

A similar expression pattern was observed in carbonic anhydrases (CA; figure 6), which are enzymes that facilitate the interconversion between CO^2^ and HCO^3-^ and are used as a CCM [12]. It plays a vital role in the calcification of various organisms, including corals, clams and coccolithophores [12,38]. In giant clams, CA2-like in mantle tissues enhance the supply of carbonic ions to the symbionts, which could boost their photosynthetic efficiency [25]. Although photosymbiosis in giant clams and Fraginae cockles has evolved independently [4], it is possible that they share a common CCM, which is prevalent in many marine organisms.

Another notable gene is the ribonucleoside-diphosphate reductase subunit M2 (RRM2), which is a small subunit ribonucleotide reductase. These enzymes facilitate the conversion of nucleoside triphosphate (NTPs) into deoxynucleoside triphosphate (dNTPs), which are crucial for DNA replication and repair [39]. dNTPs are involved in multiple immune-related pathways, including SAMHD1 virus restriction [40]. Interestingly, RRM2 has been found to be downregulated during immune stimulus in the Caribbean coral *Orbicella faveolate* [41], which suggests the involvement of RRM2 in suppressing immune responses in marine invertebrates [42], and its potential role in symbiont maintenance. In addition to RRM2, several GO terms related to the immune system were found to be downregulated in the *F. sueziense* mantle under the ambient light condition (table 2).

Of the various molecular mechanisms identified through our gene expression profiles, the innate immune system stands out as particularly interesting. It has a crucial role in symbiosis, particularly in symbiosis regulation, which includes (i) the recognition initiation of symbiosis, (ii) the maintenance of dynamic homeostasis of the symbiotic relationship and (iii) the occurrence of dysbiosis leading to bleaching [43]. During the early stage of symbiosis in Scleractinian corals, there is a tendency for the downregulation of genes associated with the immune system as the density of symbionts increases [44]. In the coral *Oculina arbuscula*, extensive enrichment of immune system BP terms is observed in aposymbiotic branches, which indicate that aposymbiotic branches display elevated immune response activity compared with symbiotic branches [45]. In *Exaiptasia*, Symbiodiniaceae actively suppresses the key immune gene NF-κB to facilitate the establishment of symbiosis [46]. In vertebrates, salamander cells affected by symbiosis do not exhibit indications of stress. Instead, several genes known to actively suppress immune responses against foreign invaders are highly expressed [47]. Fraginae cockles, despite being distantly related to cnidarians and vertebrates, use similar immune-suppression mechanisms for regulating symbiosis. This indicates that the evolutionary prevalence of photosymbiosis might not be solely attributed to the nutrient advantage but could also be influenced by the presence of shared ancestral immune-regulation mechanisms in metazoans.

### (c) The shared molecular mechanisms are likely results of parallel evolution

The identification of similar photosymbiosis molecular mechanisms across taxa provides valuable insights into its evolutionary trajectory. Parallel and convergent molecular evolution have been proposed to explain the emergence of similar phenotypes in distantly related taxa [48,49]. The key difference between the two trajectories is whether the molecular mechanisms evolved from pre-existing pathways in the ancestral lineage, in other words, whether key genes for the novel phenotypes are derived from the same ancient repertoires in their common ancestors. For example, parallel evolution can be observed in a large number of herbivorous insects, where similar amino acid substitutions in ATPα1 have been found to confer resistance to toxic cardenolides [50]. On the other hand, convergent trait evolution resulted from different genetic mechanisms found in beach mice: a single single nucleotide polymorphism (SNP) in the melanocortin-1 receptor (Mc1r) gene led to the lighter coloured coats in the Gulf Coast beach mice, while it was not discovered in the Atlantic Coast beach mouse with the similar colour pattern [51].

The similar immune regulations underlying photosymbiosis across different hosts provide evidence for parallel evolution. Although lineage-specific gene gains and losses have occurred throughout the evolutionary history of the metazoan innate immune system, many genetic components of the immune system were already present in ancestral metazoans [52]. For example, RIG-I-like receptors can detect viral pathogens and trigger antiviral responses in various animal species, and they likely originated early in the evolution of metazoans [52]. A single-cell transcriptome study also provided evidence of the deep origin of immune cell types between corals and other animal lineages [15]. Those shared key immune system genes may play a crucial role in the evolution of photosymbiosis. Under similar selection pressures, independent evolutionary processes may occur for immune-related genes. Such scenarios have been observed in plants. For example, Pik-1, an immune-related gene in rice, has independently undergone evolutionary changes from a weakly binding ancestral state to a high-affinity binding state. This adaptation enables it to specifically recognize and detect the rice blast fungus [53]. Together, our results highlight the significance of ancient gene repertoires in shaping the evolution of symbiotic relationships.

Furthermore, the importance of host immune responses may explain the scarcity of photosymbiosis in vertebrates [47]. Their adaptive immune system could retain memory of previous pathogen encounters, and enable an expedited and more effective immune response, which could prevent frequent colonization of symbionts after initial encounters by the specific antibodies [54,55]. As a result, photosymbiosis may not be viable in adult vertebrates.

It is important to acknowledge that convergent evolution of gene sequences is not unusual in nature and may also contribute to photosymbiosis evolution. The Antarctic notothenioid fishes and northern cod species, despite being phylogenetically distant, both independently evolved their respective antifreeze glycoproteins that are essential for them to survive in their freezing habitats from different ancestral sequences [56]. Another intriguing example can be found in defensins, which are a group of antimicrobial peptides produced by a diverse range of plants, animals and fungi as a defence mechanism. Its two families, *cis*-defensins and *trans*-defensins, have distinct evolutionary origins, yet they continue to exhibit similar functions [57]. Similar scenarios could apply to photosymbiosis-related genes. In the oligotrophic oceans, the nutrient advantage facilitated by photosymbiosis could lead to convergent evolution of key genes involved in essential mechanisms such as nutrient transport, symbiont regulation and immune response. However, gene annotation in non-model organisms predominantly depends on sequence similarity searches against experimentally validated sequences. Consequently, identifying convergent analogous genes that share similar functions but different sequences in non-model species remains challenging, and we could not address this aspect in this study.

Given that photosymbiosis is found in diverse metazoans and exhibits a pattern of recurrent emergence and loss, even within closely related species [4,58], both convergent and parallel evolution may be involved in the evolution of photosymbiosis. To better understand its molecular evolution, expanding our research scope beyond a few focal taxa and conducting more experimental-based research are needed.

### (d) Novel molecular mechanisms are discovered in Fraginae cockles

Evolutionary innovations in symbiotic organisms are common and enable them to occupy new ecological niches [59]. For example, symbiont-hosting organs exhibit remarkable diversity in structure and phylogenetic origin [60]. The gutless deep-sea tube worm *Riftia* uses a distinctive organ known as the trophosome to host symbiotic sulfur-oxidizing bacteria [61]. Actinorhizal plants have evolved tumorous or glandular organs that house nitrogen-fixing bacteria [62]. In Mollusca, the Bobtail squid *Euprymna scolopes* hosts the luminescent bacteria *Vibrio fischeri* in a specialized light organ, which derives from the rudiment of the embryonic hind gut-ink sac complex [63,64]. In lucinid clams, their gills undergo modifications to form a prominent and enlarged organ, providing a larger surface area for their chemosymbiotic symbionts [65]. Therefore, it is not surprising that photosymbiotic bivalves have evolved the unique tubular system for hosting symbionts [32,66].

Compared with many other metazoan hosts that harbour photosymbionts intracellularly, bivalve molluscs likely evolved novel molecular mechanisms to regulate extracellular symbionts in the tubular system. Our study revealed multiple uniquely enriched GO terms and upregulated genes that have not been previously reported in other photosymbiotic hosts, such as GO terms related to cilium motility, dynein complex and microtubules (table 3). In giant clams, it has been reported that the symbiont-hosting tubules were lined with cuboidal to low columnar cells that possess long cilia [67]. In a Fraginae species, *F. erugatum*, long and dense cilia were also found lining the tubular system [66]. It was hypothesized that the tubular system can transport symbionts to the kidneys, where some symbiont cells were hydrolyzed and subsequently expelled into the supra-branchial chamber [66]. Given the morphological observations of cilia and molecular evidence of high cilia motility, it can be inferred that bivalve hosts use cilia in the tubular system to relocate, regulate, intake or expel symbionts. Moreover, ciliary beating was found to be involved in ammonia excretion in mytilid mussels [68]. Similarly, those cilia in the symbiont-hosting tubular system could be involved in nutrient exchange between the bivalve hosts and symbionts. Further histological studies are needed to confirm the existence and locations of cilia in *F. sueziense*.

Another potential function of the upregulated ciliary activity is photodetection. In *Tridacna maxima*, the ciliary axoneme of retinal cells serves as the photoreceptive region of the eye. The ciliary structures may contribute to sensory perception and signal transduction to the optic nerve [69]. Despite the lack of evidence for complex eyes in Fraginae [69], we observed that the mantles of symbiont cockles exhibit upregulation of OPN4) genes (figure 6). OPN4 genes are known to be expressed in photosensitive tissues and encode sensory photopigments [70]. This finding suggests that even without conventional eyes, Fraginae cockles at least possess photoreceptors in their mantles, which may play a role in their ability to strategically position the symbionts in areas under the shell window structure [1], which could potentially enhance the photosynthetic efficiency of the symbionts, and ultimately facilitate energy acquisition. The discovery of photosensory in Fraginae not only provides insights into the photosymbiotic relationship but also contributes to a broader understanding of the evolution of photoreception in bivalves. It illustrates that the function of photoreception is not restricted to supporting vision. For instance, eyeless razor clams use opsins for regulating Ca^2+^- and cAMP-signalling pathways [71]. Larval oysters employ photoreception for navigation, which later evolves to regulate circadian rhythms in adulthood [72]. All evidence above suggests that photoreceptive systems in bivalves have evolved a diverse range of functions such as symbiont positioning and metabolic regulation.

Many genes that were upregulated in the baseline symbiotic state have not been reported in other photosymbiotic organisms. One such gene is Caveolin-1, which plays a role in cholesterol distribution, cell migration and signalling processes [73]. Another example is Canopy FGF Signaling Regulator 2 (CNPY2), which is known to participate in signalling pathways [74]. Although the annotation of those genes has only been conducted in model organisms such as humans, it is plausible that these genes may be co-opted to facilitate bivalve photosymbiosis (figure 6).

In addition to the genes that have been annotated and extensively studied in model organisms, a substantial proportion of genes in our transcriptomes could not be annotated. In non-model organisms that lack extensive experimental data, orthologue-based or homologue-based approaches are commonly employed. This limitation prevents the exploration of the genes that are either absent or highly divergent in the non-model organisms. Compared with the overall 44.6% unannotation rate in *F. sueziense*, the unannotation rate of those genes that are highly likely to be related to photosymbiosis is 62.4% (electronic supplementary material, table S2). These unannotated genes represent a significant knowledge gap in our understanding of genetic mechanisms of symbiosis.

Finally, some photosymbiosis mechanisms reported in other organisms are not present in Fraginae cockles. For instance, in *Exaiptasia*, it has been confirmed that carbon obtained from the symbionts plays a crucial role in facilitating the host’s ability to recycle ammonium into non-essential amino acids [16]. However, our findings indicate that there were no consistent patterns of gene expression change for some relevant genes during symbiosis disruption in *F. sueziense* (electronic supplementary material, figure S6). The absence of these mechanisms may be attributed to Fraginae hosting symbionts extracellularly. Notably, the gene LMAP1, which has been linked to symbiont phagocytosis in Cnidarians [7], is absent from the Fraginae transcriptome. While relatively more research has been conducted on cnidarian photosymbiosis, it is important to acknowledge that mechanisms identified in cnidarians may not be directly applicable to other systems. Even among closely related host lineages, certain mechanisms may not be shared. For example, genes related to light-enhanced calcification in giant clams [13] exhibit minimal expression changes under different light conditions or were not expressed at all in *F. sueziense*. This might explain why giant clams exhibit heavy shells, while Fraginae cockles use thin, semi-transparent shells to allow light penetration to the symbionts [1].

The identification of novel mechanisms within the Fraginae offers intriguing insights into the evolutionary trajectory of photosymbiosis in host organisms. Ancient gene repertoires may serve as a foundation for symbiosis-related genes, but the distinct characteristics of each host lineage may contribute to their diverse evolutionary adaptations. In corals, the symbiosome creates microenvironments within host cells, which might be an alternative of the symbiont-bearing organ to create optimal environments [75]. In single-celled eukaryotes such as foraminifera, the endosymbionts are likely to use subdivided chambers in the host, which might be a primitive form of symbiont-bearing region [76]. In comparison to other host lineages, Mollusc exhibits highly divergent organs that serve distinct functions, which allow them to evolve specific symbiont-bearing tissues. Sacoglossa sea slugs have the remarkable ability to acquire intact chloroplasts from ingested algae. These chloroplasts are then incorporated into the sea slug’s digestive gland cells, enabling them to retain functional chloroplasts within their own tissues [77]. Giant clams and Fraginae cockles host their symbionts within the tubular system derived from their digestive organs. Symbionts present in the water column are likely filtered through the gills, guiding them towards the digestive system, providing unique ways to identify and maintain symbionts [32].

## 5. Conclusion

This study sheds light on both evolutionary and mechanistic aspects of animal photosymbiosis. It demonstrates that the existence of symbionts strongly influences MF and morphological adaptation of the host. The presence of many shared molecular mechanisms between bivalves and distantly related host lineages suggests that ancient gene repertoires play important roles in the evolution of photosymbiosis and may explain the prevalence of photosymbiosis. Furthermore, novel mechanisms and the large number of unannotated photosymbiosis genes in Fraginae indicate that lineage-specific molecular tools encompass a substantial part of the host adaptation. Given the complex and diverse nature of photosymbiosis mechanisms, it is crucial for future studies to expand taxon coverage and investigate currently understudied host–symbiont systems. In addition to bioinformatics approaches, evo-devo, functional morphology and other functional validation experiments are needed for a more comprehensive investigation of molecular mechanisms underlying photosymbiosis.

## Data Availability

Electronic supplementary material is available online [78].
